# Vibrational exciton nanoimaging of phases and domains in porphyrin nanocrystals

**DOI:** 10.1073/pnas.1914172117

**Published:** 2020-03-13

**Authors:** Eric A. Muller, Thomas P. Gray, Zhou Zhou, Xinbin Cheng, Omar Khatib, Hans A. Bechtel, Markus B. Raschke

**Affiliations:** ^a^Department of Physics, University of Colorado Boulder, Boulder, CO 80309;; ^b^Department of Chemistry, University of Colorado Boulder, Boulder, CO 80309;; ^c^JILA, University of Colorado Boulder, Boulder, CO 80309;; ^d^Institute of Precision Optical Engineering, School of Physics Science and Engineering, Tongji University, Shanghai 200092, China;; ^e^Advanced Light Source Division, Lawrence Berkeley Laboratory, Berkeley, CA 94720

**Keywords:** infrared spectroscopy, molecular vibrations, scattering-scanning near-field optical microscopy (*s*-SNOM), vibrational exciton, molecular energy transport

## Abstract

Much of the electronic transport, photophysical, or biological functions of molecular materials emerge from intermolecular interactions and associated nanoscale structure and morphology. However, competing phases, defects, and disorder give rise to confinement and many-body localization of the associated wavefunction, disturbing the performance of the material. Here, we employ vibrational excitons as a sensitive local probe of intermolecular coupling in hyperspectral infrared scattering scanning near-field optical microscopy (IR *s*-SNOM) with complementary small-angle X-ray scattering to map multiscale structure from molecular coupling to long-range order. In the model organic electronic material octaethyl porphyrin ruthenium(II) carbonyl (RuOEP), we observe the evolution of competing ordered and disordered phases, in nucleation, growth, and ripening of porphyrin nanocrystals. From measurement of vibrational exciton delocalization, we identify coexistence of ordered and disordered phases in RuOEP that extend down to the molecular scale. Even when reaching a high degree of macroscopic crystallinity, identify significant local disorder with correlation lengths of only a few nanometers. This minimally invasive approach of vibrational exciton nanospectroscopy and -imaging is generally applicable to provide the molecular-level insight into photoresponse and energy transport in organic photovoltaics, electronics, or proteins.

## Significance

Molecular coupling defines fundamental properties of materials, yet spectroscopic access and imaging have remained challenging due to the short length scales of order and disorder and the low energy scales of interactions. We employ vibrational excitons as local structural probes in nanoscale infrared imaging and spectroscopy to map molecular order in a model organic electronic material (octaethyl porphyrin ruthenium(II) carbonyl). We observe coexistence of both amorphous and crystalline phases. Even for highly crystalline porphyrin, the size of individual ordered regions can remain limited to a few molecular lengths. This approach of vibrational exciton nanospectroscopy is broadly applicable to many interacting molecular systems and can provide insight into structure and energy transfer in organic electronics, proteins, or other biological complexes.

Intermolecular coupling and associated nanoscale structure are intrinsically linked to macroscopic properties from optical and electronic response to biological or catalytic function (1–5). Especially in organic molecular materials with weak intermolecular interactions and kinetically controlled structure, device performance relies on precise control of the nanoscale morphology. Optimal device performance is only achieved within a narrow range of growth parameters determined empirically due to an incomplete understanding of the low-energy interactions and resulting material properties (6–11). Indeed, low-energy intermolecular coupling of vibrations has recently emerged as an important factor for determining energy transfer on molecular length scales in organic electronics, photosynthetic systems, and proteins (12–16).

However, fundamental understanding and control of the underlying microscopic processes has remained limited due to the low-energy scales of intermolecular interactions and associated wave-function delocalization. Yet, established X-ray or electron spectroscopy and imaging for understanding atomic and molecular order with high spatial resolution rely on high-energy photons or electrons, at fluences often not compatible with delicate organic materials, and lacking sensitivity to the low-energy scales of intermolecular interactions (17–19).

Vibrational resonances as intrinsic molecular labels are uniquely sensitive to the low-energy scales of intermolecular coupling and molecular disorder. In particular, vibrational excitons can form and delocalize on nanometer-length scales (20) and can provide an exquisitely sensitive spectroscopic probe of intermolecular interactions and molecular-scale order and disorder (21–24).

**Fig. 1.**
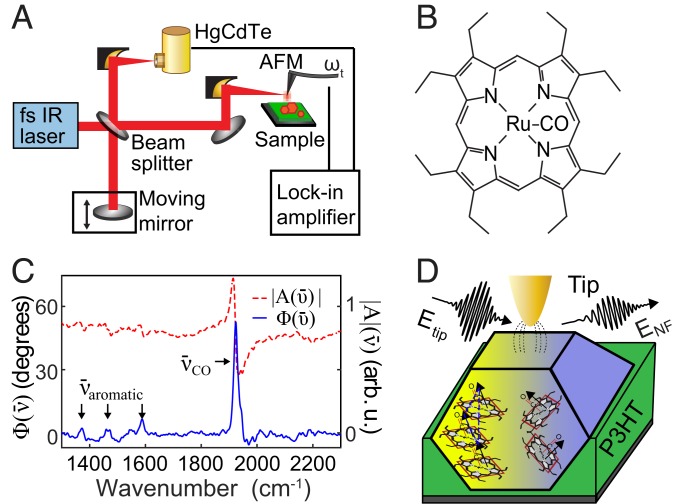
(*A*) Schematic of broadband nano-FTIR *s*-SNOM. MCT, mercury, cadmium, telluride. (*B*) Molecular structure of RuOEP. (*C*) Broadband SINS reference spectrum of pure RuOEP with 1,931-cm^−1^ metal carbonyl stretching mode ${\bar{\nu }}_{\text{CO}}$ and 1,350- to 1,600-cm^−1^ weaker aromatic modes ${\bar{\nu }}_{aromatic}$ indicated in arbitrary units (arb. u.). (*D*) Schematic of vibrational nanospectroscopy imaging of both weakly coupled RuOEP molecules (right) and strongly coupled RuOEP crystalline domains within RuOEP nanocrystals (left).

Here, we used infrared scattering-scanning near-field optical microscopy (IR *s*-SNOM) and vibrational nanospectroscopy (Fig. 1*A*) (25–30) to image vibrational excitons as a local probe of molecular order. At the example of nucleation and growth of 2,3,7,8,12,13,17,18-octaethyl-21H,23H-porphine ruthenium(II)carbonyl (RuOEP) nanocrystals (Fig. 1*B*) as a representative molecular electronic material system (31, 32), we find from nanospectroscopy and small-angle X-ray scattering (SAXS) that the porphyrin organizes with coexistence of both crystalline and amorphous phases. We measured the energy of intermolecular interactions and imaged the spatial extent of ordered and disordered regions. From coupling-induced splitting of the carbonyl stretch, we measured short-range order through delocalization of the vibrational exciton wave function on 1- to 12-nm length scales. We simultaneously imaged with 20-nm resolution heterogeneity in the degree of long-range ordering, which varies on a 50- to 150-nm length scale. Following the evolution of nanoscale heterogeneity throughout the nucleation, crystal growth, and ripening, we reveal a far more complex nanoscale phase behavior than previously assumed based on conventional spectroscopic approaches.

This approach of vibrational exciton nanospectroscopy and -imaging is generally applicable to resolve many of the molecular interactions that define the properties of the wide range of molecular electronic, photophysical molecular, or hybrid inorganic–organic materials. It complements conventional imaging and spectroscopy methods to provide a deeper fundamental understanding of low-energy intermolecular interactions, molecular assembly, and ordering at the heart of the structure–function relationship in molecular materials.

## Experiment

RuOEP aggregates were formed by phase segregation in a thin-film molecule polymer blend with a 1:10 mass ratio of regioregular poly(3-hexylthiophene-2,5-diyl) (P3HT) (Fig. 1*B*), with morphology and crystallinity controlled by solvent annealing in chloroform vapor for variable duration from 0 to 240 min (31). We monitored film quality using a combination of far-field Fourier transform infrared (FTIR), atomic force microscopy (AFM), SAXS, and synchrotron infrared nanospectroscopy (SINS) (Advanced Light Source, beamline 5.4) (25), with a representative SINS spectrum shown in Fig. 1*C*. We measured the nanoscale spectroscopic response from RuOEP aggregates using IR *s*-SNOM, as shown in Fig. 1*A*, based on a low-noise actively and passively stabilized tunable femtosecond midinfrared (mid-IR) laser source (HarmoniXX difference frequency generation [DFG], APE; Levante optical parametric oscillator [OPO], APE; Flint, Light Conversion), tuned to the frequency of the RuOEP carbonyl stretch at ∼1,930 cm^−1^, optimized with ∼100-cm^−1^ bandwidth for maximal spectral irradiance and sensitivity. As illustrated in Fig. 1*D*, we measured spatio-spectral images of both crystalline and noncrystalline regions within the RuOEP aggregates as spectrally resolved voxels with grid spacings of 20 to 50 nm. The tip-scattered near-field signal was measured interferometrically as amplitude $\left|A_{NF}\left(\bar{\nu }\right)\right|$ and phase $\Phi_{NF}\left(\bar{\nu }\right)$ spectra with 2-cm^−1^ spectral resolution by using lock-in demodulation at the second harmonic of the cantilever motion (28, 33) (for details, see Materials and Methods).

## Results

### RuOEP Aggregate Formation and Growth.

**Fig. 2.**
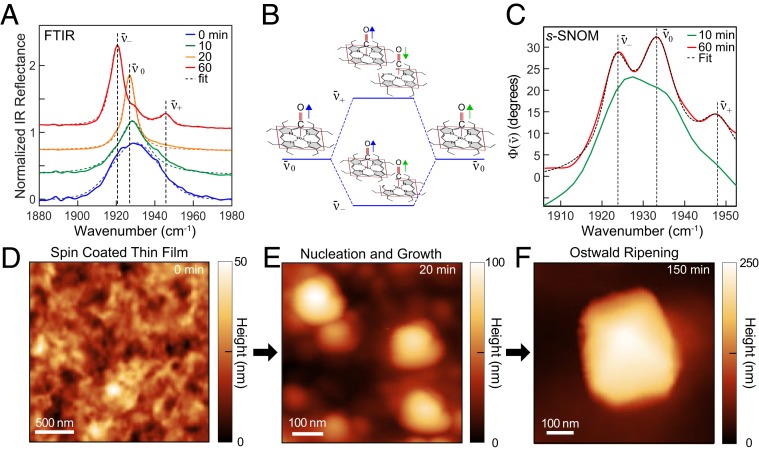
(*A*) FTIR reflectance spectra of RuOEP metal carbonyl as a function of solvent annealing after 0-min (blue), 10-min (green), 20-min (orange), and 60-min (red) chloroform vapor exposure with corresponding fits (dashed). (*B*) Schematic showing dipole coupling between neighboring molecules (green and blue arrows) inducing peak splitting of carbonyl stretch ${\bar{\nu }}_{0}$ into ${\bar{\nu }}_{-}$ and ${\bar{\nu }}_{+}$ for in-phase and out-of-phase interactions, respectively. (*C*) Representative near-field *s*-SNOM spectrum from within individual RuOEP nanocrystals after 10-min vapor annealing (green) and after 60-min annealing (red). (*D*–*F*) Evolution of morphology from AFM height images of RuOEP–P3HT blend measured as a function of solvent annealing after 0-min (*D*), 20-min (*E*), and 150-min (*F*) vapor exposure.

We first measured the ensemble-averaged vibrational response of RuOEP in P3HT thin films as a function of chloroform solvent annealing time using FTIR reflectance spectroscopy. Prior to solvent annealing, thin film samples exhibited a broad carbonyl stretch ${\bar{\nu }}_{0}$ centered at 1,931 cm^−1^ (Fig. 2*A*, blue). That peak then narrowed from an initial full-width at half-maximum of $\Gamma \left({\bar{\nu }}_{0}\right)\;=\;34\pm 4{\text{cm}}^{-1}$ (Fig. 2*A*, blue) to $\Gamma \left({\bar{\nu }}_{0}\right)\;=\;11\pm 1{\text{cm}}^{-1}$ within the first 20-min annealing time (Fig. 2*A*, orange). With further annealing, the ${\bar{\nu }}_{0}$ intensity decreased as two satellite peaks ${\bar{\nu }}_{-}$ and ${\bar{\nu }}_{+}$ (Fig. 2*A*, red) emerged. Both the larger peak, ${\bar{\nu }}_{-}\;∼\;$1,920 cm^−1^, and the smaller peak at ${\bar{\nu }}_{+}\;∼\;$1,947 cm^−1^ were spectrally narrow with $\Gamma \left({\bar{\nu }}_{\pm }\right)$ ≃ 8 to 10 cm^−1^, after 60-min vapor exposure (Fig. 2*A*, red).

Based on ensemble-averaged FTIR measurements, ${\bar{\nu }}_{0}$ was previously assigned to isolated RuOEP molecules within the P3HT matrix, while ${\bar{\nu }}_{-}$ and ${\bar{\nu }}_{+}$ were assigned to crystalline RuOEP phases separated from the P3HT (31). As shown in Fig. 2*B*, transition dipole coupling of the metal-carbonyl groups in the crystalline phase resulted in a splitting of the carbonyl stretch mode ${\bar{\nu }}_{0}$, leading to the distinct modes ${\bar{\nu }}_{-}$ and ${\bar{\nu }}_{+}$.

For the unannealed film, as shown in the AFM height image in Fig. 2*D*, we observed a uniformly disordered topography with small height variations of rms roughness  = 7 ± 1 nm. RuOEP aggregates formed already after 10 min of solvent annealing, with typical aggregate sizes of ∼100 to 200 nm, yet amorphous morphology (Fig. 2*E*). After 150-min solvent annealing, these aggregates transitioned into uniform square crystals with lateral dimensions of 200 to 500 nm and identifiable crystal faces, indicative of the emergent monoclinic crystal habit (34), as seen in Fig. 2*F*.

In complementary SAXS measurements of crystallinity, a broad peak appeared after 20-min annealing. Fits to the Scherrer equation revealed a coherence length of only 1.9±0.3nm, indicating short-range order on the two to three molecular lengths scale. With longer annealing times, the emergence of a narrow peak at the same scattering angle with continuous presence of the broad peak indicated growth of a crystalline phase with coherence length reaching ∼70±10nm for the longest annealing times of 180 min.

We next used IR *s*-SNOM to measure the spectral response from nanoscale regions within an individual RuOEP aggregate, first at early annealing times (Fig. 2*E*). Fig. 2*C* shows a representative IR *s*-SNOM spectrum $\Phi \left(\bar{\nu }\right)$ within an RuOEP aggregate solvent annealed for 10 min (green), revealing a single broad peak at 1,931 cm^−1^ with a width of ∼14 cm^−1^. Spectra for different aggregates, all annealed for the same time, were similar, with only some (±5 cm^−1^) variation in line width between aggregates. While previous far-field studies have attributed this spectrally broad feature to isolated RuOEP molecules within the P3HT matrix (31), IR *s*-SNOM instead shows that this spectral feature must be assigned to already phase-segregated RuOEP aggregates.

With further annealing, two additional peaks ${\bar{\nu }}_{-}$ and ${\bar{\nu }}_{+}$ appeared in the near-field spectra, along with ${\bar{\nu }}_{0}$, as shown in Fig. 2*C* (red), indicating phase coexistence of both a strongly coupled crystalline RuOEP alongside weakly interacting RuOEP in the same nanoscopic volumes ($8\times 10^{3}{\text{nm}}^{3}$, corresponding to $∼1\times 10^{4}$ molecules or ∼0.02 amol).

### Nanoimaging of Partially Ordered Aggregates.

**Fig. 3.**
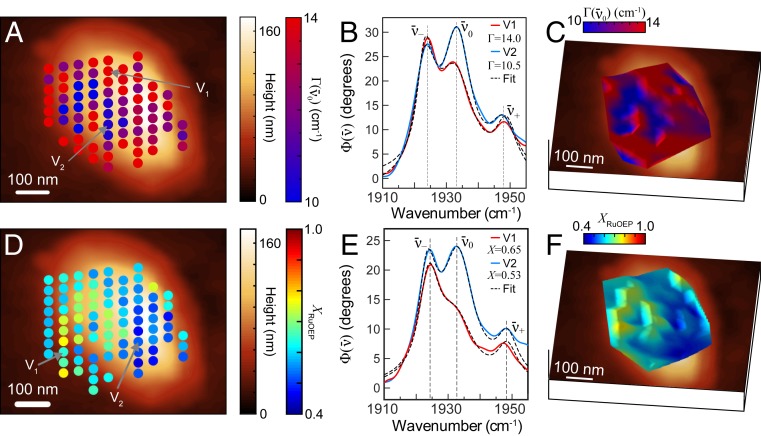
(*A*) Nanoscale map of $\Gamma \left({\bar{\nu }}_{0}\right)$ derived from fits of the voxel array at an intermediate stage of RuOEP aggregate formation. (*B*) Representative spectra and fits for locations indicated in *A*. (*C*) Corresponding 3D surface plot of $\Gamma \left({\bar{\nu }}_{0}\right)$ over the region in *A*. (*D*–*F*) Nanoscale map of crystallinity as determined by Eq. **1** and fits to the voxel arrays measured at an intermediate stage of RuOEP aggregate formation (*D*), with representative fits shown in *E* and corresponding interpolated 3D surface plots of crystallinity shown in *F*.

We next mapped the nanoscale spatial variations within RuOEP aggregates through changes in spectral broadening. Fig. 3*A* shows a map of $\Gamma \left({\bar{\nu }}_{0}\right)$ derived from fits of the voxel array across a RuOEP aggregate after a 60-min solvent anneal, with two representative spectra and fits shown in Fig. 3*B*. We found narrowed line widths $\Gamma \left({\bar{\nu }}_{0}\right)$ ranging from 10 to 14 cm^−1^ across this aggregate and typical for this annealing time. However, distinct nanoscale regions of broad and narrow line widths exist within these aggregates (Fig. 3*A*, voxels, and Fig. 3*C*, surface plot), which we attributed to spatial variations in inhomogeneous broadening.

From the IR *s*-SNOM spectra $\Phi \left(\bar{\nu }\right)$, we then derived the fraction of molecules in the crystalline phase $n\left({\bar{\nu }}_{-}\right)$ and noncrystalline phase $n\left({\bar{\nu }}_{0}\right)$. We started by determining the relative intensities $\Phi \left({\bar{\nu }}_{-}\right)$ and $\Phi \left({\bar{\nu }}_{+}\right)$, proportional to $n_{j}\cdot \mu_{j}^{2}$ for a phase *j*, from fits to $\Phi \left(\bar{\nu }\right)$ spectra using the point-dipole model (details are in *SI Appendix*). Under the approximation of uniform transition dipole moment strength of the metal-carbonyl peak in both phases (35), we can define a crystallinity index $X_{RuOEP}$:

$$X_{RuOEP}=n\left({\bar{\nu }}_{-}\right)/\left[n\left({\bar{\nu }}_{-}\right)+n\left({\bar{\nu }}_{0}\right)\right]\approx \Phi \left({\bar{\nu }}_{-}\right)/\left[\Phi \left({\bar{\nu }}_{-}\right)+\Phi \left({\bar{\nu }}_{0}\right)\right].$$ [1]

$X_{RuOEP}$ is independent of molecular orientation (29) or small variations in tip sample coupling as a result of the isotropic transition dipole moment of ${\bar{\nu }}_{-}$ and ${\bar{\nu }}_{0}$ in both phases.

We display spatially resolved nanoscale variation in crystallinity $X_{RuOEP}$ in Fig. 3*D*, with its interpolated three-dimensional (3D) representation in Fig. 3*F*. We show two spectra representative of the variation in crystallinity in Fig. 3*E*, which were selected to highlight two voxels with nearly identical response of ${\bar{\nu }}_{-}$ ($V_{1}$, red; $V_{2}$, blue), yet a stronger signal from the ${\bar{\nu }}_{0}$ mode in the $V_{2}$ spectrum, which we attributed to a lower $X_{RuOEP}$. As can be seen, the aggregate is characterized by a generally low crystallinity $X_{RuOEP}∼0.4$, with a domain of much higher crystallinity $X_{RuOEP}∼0.75$ (left center). We note the associated anticorrelation of $X_{RuOEP}$ (Fig. 3*F*) compared with $\Gamma \left({\bar{\nu }}_{0}\right)$ (Fig. 3*C*), as expected for a decrease of inhomogeneous broadening with increasing crystallinity.

**Fig. 4.**
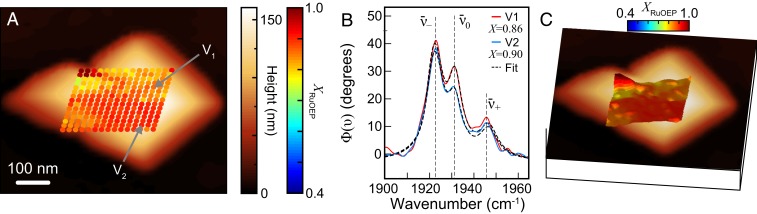
(*A*) Nanoscale map of $X_{RuOEP}$ measured with 20-nm grid spacing for a RuOEP aggregate with high crystallinity. (*B*) Representative spectra and fits for locations indicated in *A*. (*C*) Corresponding 3D surface plot of $X_{RuOEP}$ over the region in *A*.

We next imaged an RuOEP aggregate with high crystallinity. Nanoscale voxel array measured with 20-nm grid spacing (Fig. 4*A*) and corresponding surface plot (Fig. 4*C*) revealed a greater overall crystallinity $X_{RuOEP}\;∼\;$0.9 across most of the lower right region, with somewhat lower values of ∼0.75 to 0.85 across much of the upper right and the lower left. We observed variation in crystallinity across the imaged region with spatial correlation on the ∼100-nm length scale, well resolved within both the spatial resolution of *s*-SNOM determined by the ∼20-nm tip radius and the 20-nm voxel spacing.

### Intermolecular Coupling in Crystalline Regions.

**Fig. 5.**
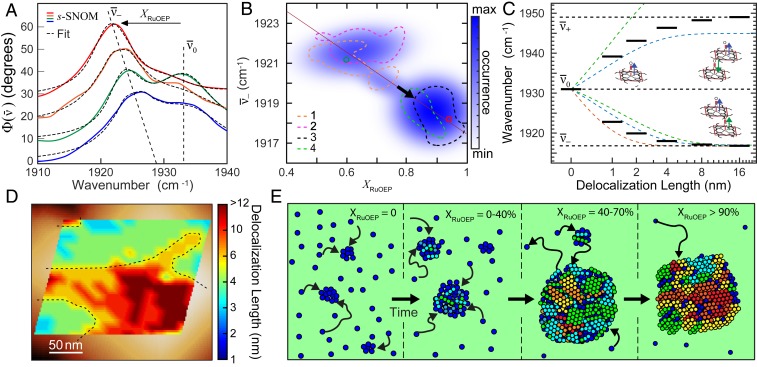
(*A*) IR *s*-SNOM spectra measured at four locations within RuOEP nanocrystals showing spectral shifts of ${\bar{\nu }}_{-}$. (*B*) Histogram of ${\bar{\nu }}_{-}$ peak center versus $X_{RuOEP}$ for voxel arrays from multiple RuOEP nanocrystals within the same sample. Max, maximum; min, minimum. (*C*) Vibrational exciton model calculation with splitting of ${\bar{\nu }}_{-}$ and ${\bar{\nu }}_{+}$ as a function of aggregate size for several models: ${1D}_{Rot}^{NN}$ (blue), ${3D}_{RuOEP}^{triclinic}$ (red), ${3D}_{RuOEP}^{tetragonal}$ (green), and ${1D}_{e\mathrm{ff}}$ (black). (*D*) Nanoimaging of crystalline domain size. Overlay shows delocalization length as determined by spectral shifts of the vibrational exciton spectra, measured with 20-nm grid spacing, with topography shown in the background. (*E*) Schematic of phase segregation during solvent annealing. Initially, seed crystals form and increase in size, followed by Ostwald ripening and associated increase in crystallinity. Dark blue circles indicate amorphous porphyrin; light blue through red circles indicate the size of crystalline domains.

We then analyzed for variations in peak position associated with crystallinity. Fig. 5*A* shows representative IR *s*-SNOM spectra in the ${\bar{\nu }}_{-}$ and ${\bar{\nu }}_{0}$ spectral region, with corresponding fits for a 60-min solvent-annealed sample. In regions with low crystallinity $X_{RuOEP}=0.28$ (blue), the peak position of ${\bar{\nu }}_{-}$ appeared near 1,924 cm^−1^. In regions with higher crystallinity $X_{RuOEP}=0.61$ (green), $X_{RuOEP}=0.74$ (orange), and $X_{RuOEP}=0.93$ (red), we observed an overall red shift in the peak position with increasing crystallinity.

Fig. 5*B* shows the corresponding 3D histogram of the variation in center frequency of ${\bar{\nu }}_{-}$ versus $X_{RuOEP}$ from statistical analysis of fits to voxel arrays from seven different nanocrystals within the same sample with moderate to high crystallinity index $X_{RuOEP}=0.40-0.99$. The 90% probability contours from four selected nanocrystals were overlaid (dashed lines), along with the results from the two individual fits from Fig. 5*A* (open circles). A correlation of red shift of ${\bar{\nu }}_{-}$ with increasing crystallinity was observed. Table 1 summarizes the general range of line-shape parameters for these and other aggregates of different crystallinity with experimental values for ${\bar{\nu }}_{-}\;≃\;$1,917 to 1,924 cm^−1^ and ${\bar{\nu }}_{+}\;≃\;$1,945 to 1,949 cm^−1^. Regions with a low crystalline fraction $X_{RuOEP}\;\leq \;0.7$ (upper left of Fig. 5*B*) have ${\bar{\nu }}_{-}$ peaks in the range 1,921 to 1,924 cm^−1^, whereas regions with high crystalline fraction $X_{RuOEP}\;\geq \;0.8$ have ${\bar{\nu }}_{-}\;≃\;$1,917 to 1,920 cm^−1^. We identified the maximum peak shift with ${\bar{\nu }}_{-}\;≃\;$1,917 cm^−1^ for highly ordered nanocrystals annealed for 180 min and the minimum peak shift with ${\bar{\nu }}_{-}\;≃\;$1,924 cm^−1^ from crystals with low $X_{RuOEP}$. The observed correlation between line shape and crystallinity thus identified nanoscale subensembles with variations in local environment, which we related to intermolecular coupling and possible finite size effects of the crystalline domains as described and modeled below.

**Table 1.** Range of vibrational line shape parameters of metal-carbonyl response within RuOEP nanocrystals as a function of crystallinity $X_{RuOEP}$ with the corresponding number of coupled molecules N as derived from the vibrational exciton model

$X_{RuOEP}$	${\bar{\nu }}_{-}$	$\Gamma \left({\bar{\nu }}_{-}\right)$	${\bar{\nu }}_{0}$	$\Gamma \left({\bar{\nu }}_{0}\right)$	${\bar{\nu }}_{+}$	$\Gamma \left({\bar{\nu }}_{+}\right)$	N*	l (nm)
0%	—	—	1,930 to 1,932	15 to 30	—	—	1	0
40 to 70%	1,920 to 1,923	6 to 9	1,930 to 1,932	11 to 15	1,945 to 1,949	6 to 12	2 to 6	1 to 5
>90%	1,917 to 1,921	5 to 10	—	—	1,945 to 1,949	6 to 12	≥6	≥5
${1D}_{e\mathrm{ff}}$ model^†^	1,917	—	1,931	—	1,949	—	∞	1.2

Spectroscopic parameters are in cm^−1^.

* *N* is the number of molecules based on 1D_eff_ model with inter molecular spacing of 1 nm.

^†^ Values ${\bar{\nu }}_{-}^{\infty }$, ${\bar{\nu }}_{+}^{\infty }$, and $l_{c}$ calculated for infinite chain with model parameters ${\bar{\nu }}_{0}=1,931$ cm^−1^ (fixed).

### Vibrational Exciton Model.

In order to determine the length scale of molecular ordering from peak splitting and spectral shifts, we modeled intermolecular coupling of the carbonyl stretch as vibrational Frenkel excitons (22, 36, 37). The dipole coupling energy ${\hat{V}}_{m,n}$ between adjacent molecules with transition dipole moments ${\vec{\mu }}_{m}$ and ${\vec{\mu }}_{n}$ and intermolecular distance $\vec{r}$ is given by (21, 36, 38, 39):

$${\hat{V}}_{m,n}=\frac{1}{4\pi \epsilon_{0}|\vec{r}\;{|}^{3}}{\vec{\mu }}_{m}\cdot {\vec{\mu }}_{n}-3\left({\vec{\mu }}_{m}\cdot \vec{r}\right)\left({\vec{\mu }}_{n}\cdot \vec{r}\right)/|\vec{r}\;{|}^{2}.$$ [2]

We then calculated the new energy eigenstates, forming the vibrational exciton band to first order in perturbation theory from the sum over all intermolecular interactions, sensitive to the number of interacting molecules and relative molecular arrangement. Based upon typical values of $\vec{\mu }\;∼\;$1 Debye and intermolecular spacing of $\vec{r}\;∼\;$1 nm, the intermolecular coupling was on the order of ${\hat{V}}_{m,m\pm 1}\;=\;$8 cm^−1^ (40, 41). This interaction strength meets the requirements of strong coupling as originally defined by Förster of $2{\hat{V}}_{m,n}>\Gamma_{vib}$ and leads to the formation of vibrational excitons, that manifests itself as a splitting of the metal-carbonyl resonance from the uncoupled value of ${\bar{\nu }}_{-}$ = 1,931 cm^−1^ into two peaks, ${\bar{\nu }}_{-}$ and ${\bar{\nu }}_{+}$ (37).

To model the evolution of ${\bar{\nu }}_{-}$ and ${\bar{\nu }}_{+}$ as a function of molecular ordering, with results summarized in Table 2 for different molecular arrangements, we first explored a simplified one-dimensional (1D) chain model with the carbonyl dipole moments ${\vec{\mu }}_{co}$ aligned parallel with respect to the molecular chain. Nearest-neighbor interactions in this 1D model (${1D}_{\|}^{NN}$) gave rise to only a single infrared (IR) active mode ${\bar{\nu }}_{-}$, with frequency progressively shifting with increasing chain length l, asymptotically approaching ${\bar{\nu }}_{-}^{\infty }\;=\;$1,917 cm^−1^ for l> 12 nm (details are in *SI Appendix*). This shift of ${\bar{\nu }}_{-}$ as a function of l can be empirically fit by using ${\bar{\nu }}_{-}\left(l\right)=\left({\bar{\nu }}_{0}-{\bar{\nu }}_{-}^{\infty }\right)\times \exp\left(l/l_{c}\right)\;+\;{\bar{\nu }}_{-}^{\infty }$ with characteristic length scale $l_{c}\;=\;$1.6 nm for a coupling constant of ${\hat{V}}_{m,m\pm 1}\;=\;$−7.1 cm^−1^. Extending this model to two molecules per unit cell, consistent with known structures of RuOEP polymorphs (40, 42), and tilting alternating carbonyl stretch ${\vec{\mu }}_{co}$ in the chain by angles θ and −θ, respectively, shifts spectral weight from ${\bar{\nu }}_{-}$ to ${\bar{\nu }}_{+}$ (${1D}_{Rot}^{NN}$). Matching the experimentally observed spectral-intensity ratio ${\bar{\nu }}_{-}/{\bar{\nu }}_{+}$, the ${1D}_{Rot}^{NN}$ results in the same values for ${\bar{\nu }}_{-}^{\infty }\;=\;$1,917 cm^−1^ and $l_{c}\;=\;$1.6 nm for $\theta \;=\;22.5^{○}$ (Fig. 5*C*, blue). However, in contrast to experiments, this model does not yet reflect the observed asymmetric splitting between ${\bar{\nu }}_{-}$ and ${\bar{\nu }}_{+}$

**Table 2.** Coupling parameters ${\hat{V}}_{m,m\pm 1}$ and predicted values of ${\bar{\nu }}_{-}^{\infty }$, ${\bar{\nu }}_{+}^{\infty }$, $l_{c}$ for different models of RuOEP



Superscripts are as follows: NN, model with nearest-neighbor interactions; tetragonal, based on structure by Miranda et al. (40); triclinic, based on structure by Salzmann et al. (42).

Although the exact crystal structure of our chloroform solvent-annealed porphyrin derivative nanocrystals is unknown, we can develop 3D exciton models for two closely related RuOEP polymorphs (40, 42) of known crystal structure to assess whether the 1D model already captures the length scale and energy shifts of intermolecular coupling associated with molecular order. Both structures have two molecules per unit cell, intermolecular spacing of 8 to 11 Å, and a mixture of positive and negative coupling constants, but differ in details of packing and molecular orientation within the unit cell. Using the triclinic structure determined by Salzmann et al. (42) and ${\hat{V}}_{m,m\pm 1}\;=\;$8.5 cm^−1^(${3D}_{RuOEP}^{triclinic}$) results in a single IR active mode ${\bar{\nu }}_{-}^{\infty }\;=\;$1,917 cm^−1^, in good agreement with experiment, and $l_{c}\;=\;$0.7 nm, but lacks an IR active ${\bar{\nu }}_{+}$ mode (Fig. 5*C*, red), like ${1D}_{\|}^{NN}$. In contrast, the tetragonal structure from Miranda et al. (40) and ${\hat{V}}_{m,m\pm 1}\;=\;$−8.3 cm^−1^ results in a ${\bar{\nu }}_{-}^{\infty }$ mode at 1,917 cm^−1^, ${\bar{\nu }}_{+}^{\infty }\;=\;$1,960 cm^−1^, and $l_{c}\;=\;$2.3 nm (${3D}_{RuOEP}^{tetragonal}$). This splitting shows ${\bar{\nu }}_{+}^{\infty }$ asymmetrically split from the uncoupled frequency, but blue-shifted by 11 cm^−1^ compared to experiment.

Comparing the 1D and 3D models, we conclude that $l_{c}$ of 12 nm and associated redshift of ${\bar{\nu }}_{-}$ is insensitive to the exact crystal structure, but the asymmetric splitting between ${\bar{\nu }}_{-}^{\infty }$ and ${\bar{\nu }}_{+}^{\infty }$ appears to depend on the details of the lattice structure, in particular, the next-nearest-neighbor interaction and its sign relative to the nearest-neighbor term. However, the characteristic length scale of the vibrational excitons shift of ${\bar{\nu }}_{-}$ being similar between 1D and 3D models suggests that an effective 1D model (${1D}_{e\mathrm{ff}}$) can adequately describe the fundamental vibrational exciton formation. With two molecules per unit cell, relative tilt of ${\vec{\mu }}_{co}$, and an additional next-nearest-neighbor term ${\hat{V}}_{m,m\pm 2}$, as characteristic for the range of experimentally known crystal structures, an effective 1D model yields a blue shift of ${\bar{\nu }}_{+}^{\infty }$ and asymmetric splitting. Specifically, for imposing a small next-nearest-neighbor coupling term ${\hat{V}}_{m,m\pm 2}\;=\;$1.0 cm^−1^, within the range of typical values for known 3D structures, this effective 1D model results in $l_{c}\;=\;$1.2 nm with splitting ${\bar{\nu }}_{-}^{\infty }\;=\;$1,917 cm^−1^ and ${\bar{\nu }}_{+}^{\infty }\;=\;$1,949 cm^−1^, in good agreement with experimental observations (Fig. 5*C*, discrete black lines).

### Growth of Nanoscale Crystalline Regions.

The peak positions ${\bar{\nu }}_{-}$ and associated values of crystallinity $X_{RuOEP}$ (Fig. 5*B*) then correspond to vibrational exciton delocalization starting at 1 to 2 nm (two to three molecules) for $X_{RuOEP}\;∼\;0.4$, reaching ≥10 nm (∼ 11 molecules) for $X_{RuOEP}\;∼\;0.9$ We further compared the spectroscopic measurement of vibrational exciton delocalization to SAXS measurements of crystallinity. In the most highly crystalline samples annealed for 180 min, we found that the average domain size in crystalline regions reached 70±10 nm. From these highly annealed nanocrystals, we identified the maximum peak shift in IR *s*-SNOM spectra ${\bar{\nu }}_{-}$ = 1,917 cm^−1^, which is the asymptotic limit of spectral shifts for delocalized vibrational excitons ≥10 nm. Similarly, we identified the minimum peak shift of ${\bar{\nu }}_{-}\;=\;$1,924 cm^−1^ from aggregates with low $X_{RuOEP}$, in agreement with the only short-range order spanning two to three molecules observed in SAXS measurements for nanocrystals with short annealing times. We then used experimental values of ${\bar{\nu }}_{-}$ to create a corresponding map of exciton delocalization, as shown in Fig. 5*D* for the RuOEP aggregate from Fig. 4*A*. We found that excitons were confined to only 2 to 4 nm in some regions (blue and green shading), whereas the vibrational excitons were more delocalized across 6 to 12 nm in other regions (yellow and red) (separated by dashed lines as a guide to the eye).

## Discussion

In the following, we discuss our observations of coexistence of ordered and disordered phases within RuOEP using vibrational excitons as an intrinsic and structurally sensitive label in IR *s*-SNOM imaging. These crystalline and disordered phases coexist, even within the nanoscale ∼ 20-nm near-field probe volume. We indeed confirmed by SAXS both the existence of ordered and disordered phases and the increase in average domain size. In contrast, earlier studies using conventional spectroscopies that lack morphological information have assumed that the ordered and disordered phases exist in morphologically distinct and spatially separate regions, with the ordered phase in nanocrystals and disordered phase within the P3HT matrix (31).

Our nanospectroscopy showed that, although the crystallinity index and vibrational exciton delocalization were overall correlated as could be expected, crystallite domain size remained small until the crystallinity index was very high, and significant short-range disorder remained, even with a high degree of long-range order. The observed aggregate ripening, increase in crystallinity, and increasing crystalline domain size are likely associated with Ostwald stages of growth, in which an amorphous phase is favored at early times to be replaced by an increasing fraction of a crystalline phase at longer annealing times (43–47). As illustrated in Fig. 5*E* (first and second panels), nanocrystals formed and initially grew quickly. Yet, even large nanocrystals (third panel) still contained primarily the disordered phase, and only later stages (fourth panel) exhibited an increasing fraction of the ordered phase, but still contained a variable degree of local disorder.

This nanoscale- and molecular-level insight into the presence of disordered and ordered phases and vibrational excitons confined to few-nanometer domains can provide critical understanding for the design of functional materials. Both crystalline and amorphous phases may occur in devices made with porphyrins or other molecular materials, and tradeoffs exist between benefits of crystalline phases with typically higher charge carrier mobility and exciton diffusion length versus amorphous phases that may exhibit improved interfacial miscibility and improved charge separation (48–51). The spatial organization of domains remains poorly understood across multiple length scales and is often only characterized by ensemble-averaged measurements due to the combined challenge of short length scales and low-energy scales of interaction. X-ray nanoimaging and transmission electron microscopy (TEM) methods are poorly suited to molecular materials due to low-scattering cross-sections and low damage threshold. Even specialized low-dose imaging has yet been unable to distinguish adjacent amorphous and crystalline phases (52). While previous studies using TEM and far-field spectroscopy had assigned broad spectral features to isolated RuOEP with the P3HT matrix (31), we instead observed, using vibrational exciton nanospectroscopy, the coexistence of amorphous and crystalline phases, with domains as small as a few molecules. We found molecular crystals exhibiting both long-range order, as determined by sharp SAXS diffraction peaks, yet with amorphous phase material in the same sample. Albeit a near-surface probe, the IR *s*-SNOM spectroscopic depth of ∼20 nm is well suited to investigate a wide range of functional materials and organic electronics that commonly exhibit both few-nanometer crystalline domains and spatial heterogeneity on multiple length scales (53).

Spectroscopic measurements of vibrational mode coupling in molecular aggregates can add understanding of fundamental physical properties of vibrational excitons and their spatial delocalization. The vibrational exciton is a delocalized, collective vibration that exhibits phonon softening and increase of splitting with increasing delocalization. However, lattice disorder in heterogeneity in coupling strengths can lead to localization of the vibrational exciton, in addition to the confinement we model (16). Additionally, thermal fluctuations can lead to either self-trapping or Anderson localization, depending on temperature (12, 54). These localization phenomena remain broadly challenging, both theoretically and experimentally, and temperature-dependent IR *s*-SNOM combined with high-resolution structural measurements could add fundamental understanding of energy transport in coupled molecular systems.

In conclusion, we used IR *s*-SNOM nanospectroscopy of a vibrational exciton as a probe to image from local molecular order to nanoscale crystallinity. We measured coexistence of both ordered and disordered phases in RuOEP within the same morphological region. From spectroscopic signatures of vibrational exciton delocalization, we observed that spatial heterogeneity extends down to molecular scales. We found molecular crystals with long-range order and sharp SAXS diffraction peaks, yet with nanometer-scale coexistence of amorphous and crystalline phases with domains as small as a few molecules. Our approach of nanospectroscopy of coupled vibrational modes is readily generalizable and can provide fundamental insight into few-molecule to long-range order that determines charge, thermal, and energy transport.

## Materials and Methods

### Sample Preparation.

A 1:10 mass ratio blend of porphyrin–polymer was prepared and mixed in a 1 wt% solution of 1.5 mL of chloroform and given 24 h to dissolve following sonication. Commercial P3HT and RuOEP were acquired from Rieke Metals Inc. and Sigma-Aldrich, respectively. Thin films were prepared by spin-coating from solution at 3,000 rpm for 1 min onto freshly prepared, template-stripped Au substrates. The thin films were subsequently solvent-annealed in chloroform vapor for up to 240 min.

### Far-Field FTIR and SAXS.

Far-field reflectance data were acquired by using a commercial FTIR spectrometer (Vertex v70; Bruker Optics) coupled to an IR microscope (Hyperion; Bruker Optics) and a water-cooled SiC globar source. The microscope aperture was set to ∼100 μm, using a 32× Schwarzschild objective. Two hundred sample scans were averaged and normalized to an Au reference, with 2-cm^−1^ spectral resolution and 2-min acquisition time per sample measurement. Sample measurements were performed in various spatial locations for each annealed film, to ensure consistency of ensemble-averaged spectra. SAXS was performed on solvent-annealed films by using a Bruker Nano/Microstar instrument with a copper source λ = 1.54 Å.

### Mid-IR Generation and IR *s*-SNOM.

Tunable mid-IR light was generated by DFG of signal and idler beams (HarmoniXX DFG, APE) from a femtosecond OPO (Levante OPO, APE) pumped by a low-noise Yb oscillator operating at 75.7 MHz, with a pulse duration of 93 fs and an average power of 6 W (Flint, Light Conversion). The DFG light was tunable from 4 μm (2,500 cm^−1^) to 15 μm (666 cm^−1^), with a pulse duration of 150 fs. The laser-output frequency was tuned to that of the carbonyl vibration, with a bandwidth of 100 cm^−1^. Sixteen milliwatts of DFG light was attenuated by 75% with a mesh filter and was directed into the *s*-SNOM instrument (nanoIR2-s prototype, Anasys Instruments/Bruker) as described previously (55). Active and passive stabilization of the pump laser and OPO stabilized the DFG and beam pointing, which enabled low-noise operation and stable spectral output that is required for the high signal-to-noise ratio and high spectral resolution necessary for the measurements.

Incident laser light was focused by an off-axis parabolic mirror (numerical aperture = 0.45, f = 25.4 mm) onto the metallized tip (160AC-GG OPUS, MikroMasch) of an atomic force microscope (nanoIR2-s prototype, Anasys Instruments/Bruker) operating in intermittent contact (tapping) mode (56). The near-field tip-scattered signal was detected interferometrically in a backscattering geometry by an HgCdTe detector (MCT KLD-0.5-J1/DC/11, Kolmar Technologies) using lock-in demodulation (Zurich Instruments HF2LI) at the second harmonic of the cantilever motion. Complex valued Fourier transform near-field spectra were represented as amplitude $A_{NF}\left(\bar{\nu }\right)$ and phase $\Phi_{NF}\left(\bar{\nu }\right)$, containing information about the dispersive and absorptive components of the IR response, respectively (28, 33). IR *s*-SNOM spectra were collected with 2-cm^−1^ spectral resolution by using rapid-scan detection and averaging times of 1 to 4 min.

### Data Availability.

The procedures of experiments and simulations are described in detail in Materials and Methods and in *SI Appendix*. Data, computer code for fitting, and modeling code are available through the Open Science Repository (https://osf.io/72vcx/) (57).

## Supplementary Material

Supplementary File
