# Supplementary material for: Vibrational exciton nanoimaging of phases and domains in porphyrin nanocrystals
Source: Proc Natl Acad Sci U S A. 2020 Mar 13;117(13):7030–7. doi: 10.1073/pnas.1914172117 (PMC7132254; doi:10.1073/pnas.1914172117)
Supplement: Supplementary File [file pnas.1914172117.sapp.pdf]

# Supplementary Information for

## Vibrational exciton nano-spectroscopic imaging of molecular coupling and disorder in functional molecular materials

**[a,b]Eric A. Muller,[a]Thomas P. Gray,[c]Zhou Zhou,[c]Xinbin Cheng,**
**[a,d]Omar Khatib, [d]Hans A. Bechtel,[a]Markus B. Raschke**[1]

[a]Department of Physics, Department of Chemistry, and JILA, University of Colorado, Boulder, CO 80309; [b]Present Address: Department of Chemistry, Colgate University, Hamilton, NY 13346; [c]Institute of Precision Optical Engineering, School of Physics Science and Engineering, Tongji University, Shanghai, China, 200092; [d]Advanced Light Source Division, Lawrence Berkeley Laboratory, Berkeley, CA 94720

[1]To whom correspondence should be addressed.
E-mail: markus.raschke@colorado.edu and emuller@colgate.edu

### This PDF file includes:

Supplementary text
Figs. S1 to S4
SI References

## Supporting Information Text

## Vibrational Exciton Model

Here we provide additional details of the vibrational exciton models used in the main text. We begin with a one exciton Hamiltonian for coupling between $N$ molecules with open boundary conditions. This can be expressed as a matrix:

$$\widehat{H} = \begin{bmatrix} \bar{\nu}_1 & V_{1,2} & V_{1,3} & \cdots & V_{1,N} \\ V_{1,2} & \bar{\nu}_2 & V_{2,3} & & \\ V_{1,3} & V_{1,3} & \bar{\nu}_3 & & \\ \vdots & & & \ddots & \\ V_{N,1} & \dots & & & \bar{\nu}_N \end{bmatrix} \quad [1]$$

where diagonal values $\bar{\nu}_n$ are the ground state energy $\bar{\nu}_0$ =1931 cm$^{-1}$ and off diagonal interaction terms $V_{m,n}$ between any two molecules, with $\vec{\mu}_m$ and $\vec{\mu}_n$ is given by equation 2 of the main text. We solve for the resulting eigenvalues and eigenvectors describing how each atom moves during a specific excitation. The IR response of each mode was inferred from the relative $\vec{\mu}(\bar{\nu})^2$ obtained from the sum over each eigenvector weighted by molecular orientation (1). To determine the size dependent effect on $\bar{\nu}_-$ and $\bar{\nu}_+$, 1D models were explored for chain lengths from 1 to 50 molecules, and 3D models for parallelepipeds 1 to 15 molecules on each side.

We first show the 1D model with a single molecule per unit cell $1\mathrm{D}_{||}^{\mathrm{NN}}$ and 1D model with two molecules per unit cell and a rotation between alternating molecules $1\mathrm{D}_{\mathrm{Rot}}^{\mathrm{NN}}$, both shown in Fig. S1 (light blue, black). In both models, we use an intermolecular separation of 1 nm consistent with the typical intermolecular spacing in experimentally determined crystal structures of RuOEP (2, 3). Both models include the nearest neighbor coupling term $V_{m,m\pm1}$. The coupling value used, as well as resulting $\bar{\nu}_-^{\infty}$, $\bar{\nu}_+^{\infty}$ and $l_c$ can be seen in Table 2 of the main text. The nearest neighbor interaction $V_{m,m\pm1}$ is stronger than the next nearest neighbor term $V_{m,m\pm2}$ by a factor of 8, with other terms getting progressively weaker. As such, it has the strongest impact with other terms introducing small corrections. Including next nearest neighbor interactions $V_{m,m\pm2}$ into a 1D model ($1\mathrm{D}_{\mathrm{Rot}}^{\mathrm{NNN}}$) redshifts $\bar{\nu}_+^{\infty}$ by 2 cm$^{-1}$ (Fig. S1 brown) compared to the $1D_{Rot}^{NN}$ case, in the opposite direction as experiment. The crystal structure of RuOEP nanocrystals is solvent dependent (2, 3). In the absence of a known crystal structure for the nano-crystals, two closely related polymorphs of RuOEP were chosen to expand into a 3D vibrational exciton model. For the two 3D models (Fig. S1 dark blue, red), molecular spacing and orientation were determined from the chosen structures (2, 3), we include all short- and long-range intermolecular interactions $V_{m,n}$ across the parallelepipeds.

We next compare the strength of the upper and lower branches of the exciton peak. Each branch forms a band of allowed states, so we explicitly calculate the strength of possible optical transitions. For $1\mathrm{D}_{||}^{\mathrm{NN}}$, greater than 80% of $\vec{\mu}(\bar{\nu})^2$ is concentrated into only the lowest energy exciton state, with $> 95\%$ contained in modes that are within 2 cm$^{-1}$ of the lowest energy mode. For all models, a weighted average of $\bar{\nu} * \vec{\mu}(\bar{\nu})^2$ is performed to determine $\bar{\nu}_-$ and when applicable $\bar{\nu}_+$. By rotating alternating molecules by $\theta$, $-\theta$ respectfully in $1\mathrm{D}_{\mathrm{Rot}}^{\mathrm{NN}}$ some of $\vec{\mu}(\bar{\nu})^2$ is transferred from $\bar{\nu}_-$ to the highest eigenvalue forming $\bar{\nu}_+$. For $\theta$ =22.5 degree, $\approx 15\%$ of $\vec{\mu}(\bar{\nu})^2$ is transferred to $\bar{\nu}_+$. This ratio of $\bar{\nu}_-$ to $\bar{\nu}_-$ approximately matches observed ratios as can be seen in figure S2 C.

## Spectral Fitting and Crystallinity Index

In the linear infrared spectroscopy response, the intensity of the peak in the vibrational spectrum is proportional to the total number of molecules in the respective ordered and disordered phases and the square of the corresponding transition dipole moment. In order to calculate the optical response of RuOEP, we first determine the relative transition dipoles of each vibrational mode, where the transition probability of the mode is proportional to $n_j \cdot \vec{\mu}_j^2$ for $n$ molecules in phase $j$ and


[a,b]Eric A. Muller,[a]Thomas P. Gray,[c]Zhou Zhou,[c]Xinbin Cheng,
[a,d]Omar Khatib, [d]Hans A. Bechtel,[a]Markus B. Raschke

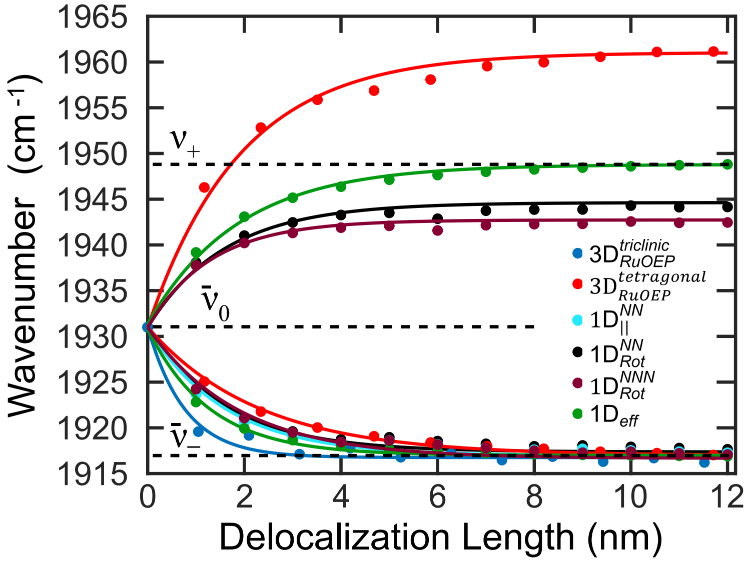


**Fig. S1.** Relative splitting of $\bar{\nu}_-$ and $\bar{\nu}_+$ for several different vibrational exciton models demonstrating that while splitting between $\bar{\nu}_-$ and $\bar{\nu}_+$ is sensitive to the model, the length scale $l_c$ over which the splitting occurs is insensitive to the details of molecular orientation. Parameters and results of each model are summarized in Table 2 of the main text.

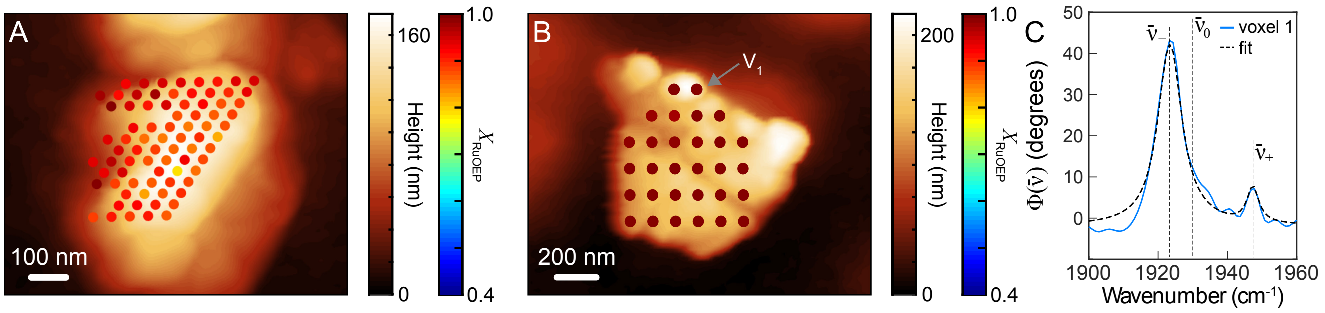


**Fig. S2.** Nanoscale maps of crystallinity measured at an intermediate stage of RuOEP aggregate formation. Fits to the voxel arrays are determined by Eq. 1 of the main text.



[a,b]Eric A. Muller,[a]Thomas P. Gray,[c]Zhou Zhou,[c]Xinbin Cheng,
[a,d]Omar Khatib, [d]Hans A. Bechtel,[a]Markus B. Raschke

transition dipole of that vibrational mode $\vec{\mu}_j$. In each of the models, dipole coupling causes splitting of the $\bar{\nu}_0$ peak into both the $\bar{\nu}_-$ and $\bar{\nu}_+$. The total transition dipole moment is conserved upon vibrational exciton formation, such that $\vec{\mu}_0 = \vec{\mu}_- + \vec{\mu}_+$ (1). In the simplest 1D model, the upper branch is symmetry forbidden so that $\vec{\mu}_0 = \vec{\mu}_-$. In our observations, the peak associated with $\vec{\mu}_+$ indicates a weakly allowed transition, such that the approximation $(\vec{\mu}_0)^2 \simeq (\vec{\mu}_-)^2$ is valid. Because the intensity of the two peaks are proportional to the molecules in each phase multiplied by the transition dipole moment and the tip-sample coupling, the ratio between intensities of the two peaks can be related to the ratio of molecules in each phase.

We then calculate the optical response of the RuOEP with a Lorentzian oscillator model. The frequency dependent infrared dielectric constant $\epsilon_{sample}(\bar{\nu})$ for RuOEP can be expressed as the sum of individual molecular vibrations:

$$\epsilon_{sample}(\bar{\nu}) = \epsilon_0 + \sum_j N/V \cdot cos^2(\frac{\delta\vec{\mu}_\alpha^j}{\delta Q_j})\frac{\bar{\nu}_j^2}{\bar{\nu}_j^2 - \bar{\nu}^2 - 2i\bar{\nu}\gamma_j} \qquad [2]$$

for a material with density $N/V$ and zero-frequency dielectric constant $\epsilon_0$. Each vibrational mode $j$ with transition dipole $\delta\vec{\mu}_\alpha^j/\delta Q_j$ contributes Lorentzian response with center frequency $\bar{\nu}_j$ and damping coefficient $\gamma_j$ (4). For RuOEP, we include the number of oscillators contributing to each vibrational mode with $\bar{\nu}_0$ of the disordered phase and with $\bar{\nu}_-$ and $\bar{\nu}_+$ for the ordered phase. The number of molecules in the ordered versus disordered phase and the dipole moment of each mode then each contribute to the overall wavenumber dependent dielectric response calculated for RuOEP.

We model the tip interaction using a spherical dipole model. The frequency dependent infrared dielectric constant of the sample $\epsilon_{sample}(\bar{\nu})$ can be expressed as the sum of individual molecular vibrations, shown in equation 3, for a material with density $N/V$ and zero-frequency dielectric constant $\epsilon_0$. Each vibrational mode $j$ with transition dipole $\delta\mu_\alpha^j/\delta Q_j$ contributes Lorentzian response with center frequency $\bar{\nu}_j$ and damping coefficient $\gamma_j$.

The polarizability of the *s*-SNOM tip is approximated as that of a polarizable sphere with radius $R$ and dielectric constant $\epsilon_{tip}$ as given by the Claussius-Mossetti equation $\alpha_{sph} = 4\pi R^3(\epsilon_{tip} - 1)/(\epsilon_{tip} + 2)$. The scattered signal from the near-field is then proportional to the effective polarizability of the tip and sample:

$$\alpha_{eff} = \alpha_{sph}\left[1 - \frac{\alpha_{sph}}{16\pi(R_t + z)^3}\frac{\epsilon_{sample} - 1}{\epsilon_{sample} + 1}\right]^{-1} \qquad [3]$$

for a height $z$ above the sample. We determine the polarizability of the tip using Equation 3, coupled through near-field interactions to the sample with $\epsilon_{sample}(\bar{\nu})$ given by Equation 2. We then calculate the *s*-SNOM signal as the scattered optical field and include both modulation of the tip and subsequent lock-in detection through the distance dependence of the polarizability. The calculated scattered infrared *s*-SNOM signal is then dependent upon the number of RuOEP molecules in crystalline or amorphous phase within the probed volume through the oscillator strength of the $\bar{\nu}_-$, $\bar{\nu}_+$, and $\bar{\nu}_0$ modes. We use the oscillator strength, peak, position, and line width as free parameters in an automated fitting routine. Points with spectral intensity or fit quality below a set threshold are automatically removed.

## Nano-Imaging of Vibrational Exciton Probes

Figures 3-5 of the main text show several crystallites with crystallinity ranging primarily between $X_{\mathrm{RuOEP}} = 0.4 - 0.95$. We show in Figure S2A an additional aggregate with higher crystallinity of $X_{\mathrm{RuOEP}} = 0.85 - 0.95$, with some variation in crystallinity across the measured region. Additionally, in Figure S2B we show an aggregate with crystallinity of 0.95-0.99 across the entire measured region. Figure S2C shows a fit to a representative spectrum with high crystallinity, measured at the voxel location $V_1$ in Fig. S2B.

## Statistical Analysis

We analyze statistical variation in the spectral response of $\Gamma(\bar{\nu}_-)$ and $\bar{\nu}_-$ at each voxel from measurements of many RuOEP nano-crystals. 414 voxels, with fit quality above a set threshold, were used with kernel density estimation to make 2D histograms

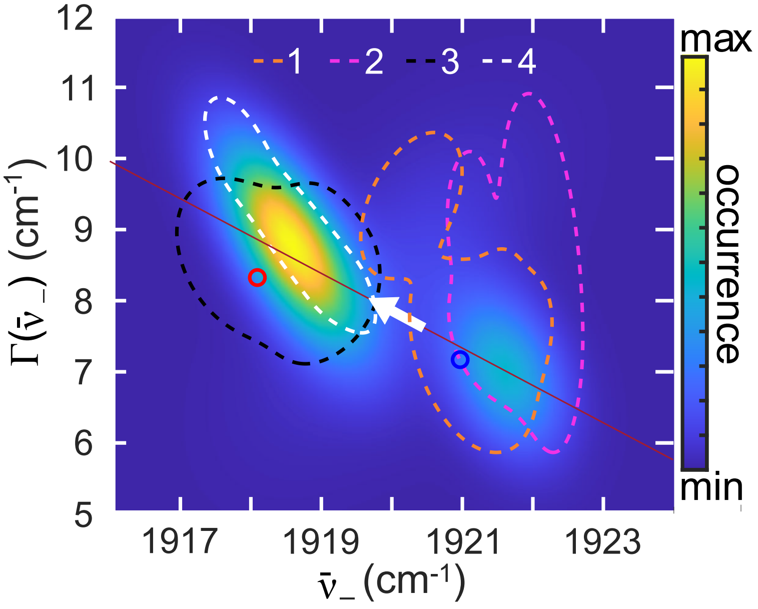


**Fig. S3.** Statistical analysis of spectroscopic response from voxel arrays measured across multiple RuOEP nano-crystals showing $\Gamma(\bar{\nu}_-)$ vs. $\bar{\nu}_-$ peak center.



[a,b]Eric A. Muller,[a]Thomas P. Gray,[c]Zhou Zhou,[c]Xinbin Cheng,
[a,d]Omar Khatib, [d]Hans A. Bechtel,[a]Markus B. Raschke

of $X_{\mathrm{RuOEP}}$ vs, $\bar{\nu}_-$ (main text Fig. 5c) and $\bar{\nu}_-$ vs $\Gamma(\bar{\nu}_-)$ (Fig. S3). In addition, main text Fig. 5c was plotted on a log scale to deemphasize higher concentrations at higher crystallinity due to more crystals being measured in this regime. We observe in Figure S3 only a weak trend towards spectral broadening correlated with the red-shift of $\bar{\nu}_-$. We note that either broadening or narrowing could be expected by e.g. exchange narrowing or access to new dephasing pathways could be expected, and we are unable to distinguish these.

## Small Angle X-ray Scattering

We perform structural characterization of RuOEP nano-crystals using SAXS. We perform SAXS using a copper X-ray source on RuOEP/P3HT spin-coated as thin films on Si substrates and subsequently solvent annealed in chloroform for up to 180 minutes (Fig. S4). For short annealing times, we observe a single broad peak in the SAXS at a spacing of 1.55 nm, assigned to the RuOEP unit cell, becoming a sharp peak as annealing times are increased, as seen in Figure S4A-B. Fits to the area integrated spectra shows a line width of $0.56 \pm 0.05$ nm for the 20 min annealed sample, decreasing slightly to $0.51 \pm 0.05$ nm at 60 min. As annealing time is increased, a narrow second peak appears at the same location, which we assign to increasing presence of the crystalline phase. We fit this to the sum of two gaussians, finding line widths of $0.47 \pm 0.05$ nm and $0.016 \pm 0.002$ nm. Using the Scherrer equation, we measure the size of these two populations to be $2.1 \pm 0.3$ nm and $70 \pm 10$ nm.

## References


1. Peter Hamm and Martin Zanni. *Concepts and methods of 2D infrared spectroscopy*, volume 9781107000. Cambridge University Press, 2011.
2. Renzo Salzmann, Michael T. McMahon, Nathalie Godbout, Lori K. Sanders, Mark Wojdelski, and Eric Oldfield. Solid-State NMR, Crystallographic and Density Functional Theory Investigation of Fe-CO and Fe-CO Analogue Metalloporphyrins and Metalloproteins. *Journal of the American Chemical Society*, 121(16):3818–3828, 1999.
3. Katrina M. Miranda, Xianhui Bu, Ivan Lorković, and Peter C. Ford. Synthesis and Structural Characterization of Several Ruthenium Porphyrin Nitrosyl Complexes. *Inorganic Chemistry*, 36(21):4838–4848, 1997.
4. Reinhard Scholz, Marion Friedrich, Georgeta Salvan, Thorsten U Kampen, Dietrich R T Zahn, and Thomas Frauenheim. Infrared spectroscopic study of the morphology of 3,4,9,10-perylene tetracarboxylic dianhydride films grown on H-passivated Si(111). *J. Phys. Condens. Matter*, 15(38):2647–2663, 2003.

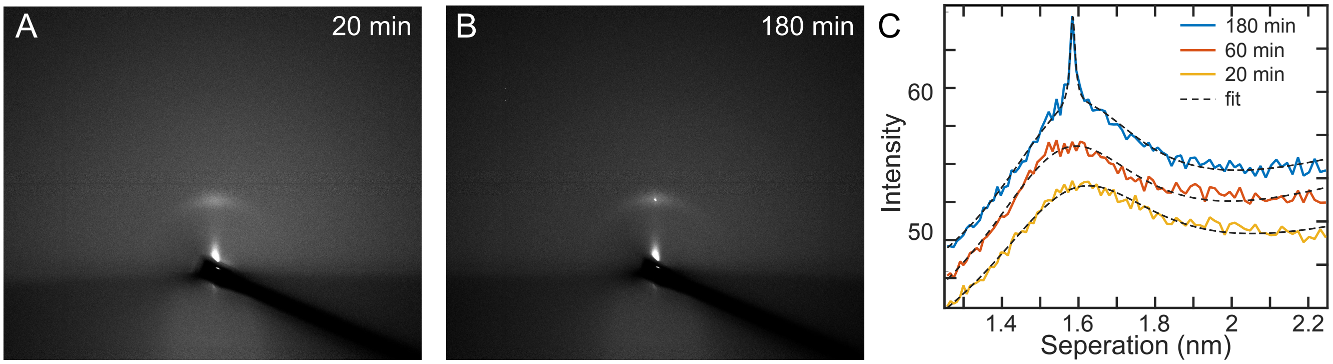


**Fig. S4.** X-ray diffraction spectra of RuOEP solvent annealed for (A) 20 minutes and (B) 180 minutes. (C) X-ray diffraction pattern for RuOEP with fits to the Scherrer equation. The 180 minute spectrum is fit to the sum of two gaussians corresponding to the crystalline and amorphous populations.


[a,b]Eric A. Muller,[a]Thomas P. Gray,[c]Zhou Zhou,[c]Xinbin Cheng,
[a,d]Omar Khatib, [d]Hans A. Bechtel,[a]Markus B. Raschke
